# The Crystal Structure of *Giardia duodenalis* 14-3-3 in the Apo Form: When Protein Post-Translational Modifications Make the Difference

**DOI:** 10.1371/journal.pone.0092902

**Published:** 2014-03-21

**Authors:** Annarita Fiorillo, Daniele di Marino, Lucia Bertuccini, Allegra Via, Edoardo Pozio, Serena Camerini, Andrea Ilari, Marco Lalle

**Affiliations:** 1 Department of Biochemical Sciences “A. Rossi-Fanelli”, University of Rome “Sapienza”, Rome, Italy; 2 Institute of Molecular Biology and Pathology, CNR, Rome, Italy and Institute Pasteur Cenci-Bolognetti Foundation at Department of Biochemical Sciences “A. Rossi-Fanelli”, University of Rome “Sapienza”, Rome, Italy; 3 Department of Physics, University of Rome “Sapienza”, Rome, Italy; 4 Department of Health and Technology, Istituto Superiore di Sanità, Rome, Italy; 5 Department of Infectious, Parasitic and Immunomediated Diseases, Istituto Superiore di Sanità, Rome, Italy; 6 Department of Cell Biology and Neurosciences, Istituto Superiore di Sanità, Rome, Italy; Monash University, Australia

## Abstract

The 14-3-3s are a family of dimeric evolutionary conserved pSer/pThr binding proteins that play a key role in multiple biological processes by interacting with a plethora of client proteins. *Giardia duodenalis* is a flagellated protozoan that affects millions of people worldwide causing an acute and chronic diarrheal disease. The single giardial 14-3-3 isoform (g14-3-3), unique in the 14-3-3 family, needs the constitutive phosphorylation of Thr214 and the polyglycylation of its C-terminus to be fully functional *in vivo*. Alteration of the phosphorylation and polyglycylation status affects the parasite differentiation into the cyst stage. To further investigate the role of these post-translational modifications, the crystal structure of the g14-3-3 was solved in the unmodified apo form. Oligomers of g14-3-3 were observed due to domain swapping events at the protein C-terminus. The formation of filaments was supported by TEM. Mutational analysis, in combination with native PAGE and chemical cross-linking, proved that polyglycylation prevents oligomerization. *In silico* phosphorylation and molecular dynamics simulations supported a structural role for the phosphorylation of Thr214 in promoting target binding. Our findings highlight unique structural features of g14-3-3 opening novel perspectives on the evolutionary history of this protein family and envisaging the possibility to develop anti-giardial drugs targeting g14-3-3.

## Introduction

Eukaryotic 14-3-3s are a family of dimeric highly conserved proteins (∼30 kDa) with pSer/pThr binding property. When 14-3-3s are present in multiple isoforms (e.g. seven in *Homo sapiens*) they can form either homo- and/or heterodimers. Mammals, including *Homo sapiens*, contain seven 14-3-3 genes (i.e., β, γ, ε, σ, ζ, τ, η), and 15 genes coding for 14-3-3s have been identified in the plant *Arabidopsis thaliana.* [1]–[2]. 14-3-3s bind to hundreds of client proteins thus affecting many cellular processes, including metabolism, signal transduction, cell cycle, differentiation, apoptosis, protein trafficking, transcription, stress responses and malignant transformation [3]. The overall 14-3-3 architecture is well conserved among the different isoforms and eukaryotic kingdoms. Each 14-3-3 monomer consists of nine antiparallel α-helices (A-I) organized in a cup-like shape with the dimerization domain located in the N-terminal half [4]–[8]. The 14-3-3-target interaction is mediated by conserved residues in an amphipathic groove of each monomer (formed by α-helices C, E, G, and I) and requires specific pSer/pThr containing motifs on the targets. The phosphate moiety of the target binding peptide directly contacts a cluster of positively charged residues in the 14-3-3 amphipathic groove including Lys49, Arg56, Arg127 and Tyr128 (human 14-3-3ζ numbering), whereas the phosphopeptide backbone and side chains establish interactions with a hydrophobic patch of 14-3-3 conserved residues. Three general 14-3-3 consensus binding motifs have been identified: RSX(pS/pT)XP, called mode-1, where pS/pT are phosphorylated serine or threonine residues and X stands for any type of residue; RX(Y/F)X(pS/pT)XP (mode-2) [9]; and Xp(S/T)X_1-2_-COOH (mode-3), in which the phosphorylated residue occupies the penultimate C-terminal position in the target protein [10]. Nevertheless, 14-3-3s may also interact with some non-phosphorylated peptides, such as R18, containing the WLDLE sequence [11]. The overall rigid structure of the 14-3-3 dimer can induce, upon binding, conformational changes in the target protein resulting in the inter- and/or intra-compartmental sequestration of the target itself, activation/inactivation of the target enzymatic activity, and, in few cases, promotion/inhibition of the interaction between the target and other proteins [8]. From an evolutionary point of view, 14-3-3s can be subdivided into four major groups coinciding with the four kingdoms. The existence of an ancestral 14-3-3 that evolved independently into different isoforms in each kingdom has been proposed. In particular, the independent clustering of Metazoan epsilon isoforms strongly suggesting that the epsilon isoforms are relatively conserved and similar to the original animal 14-3-3 ancestor [1].

*Giardia duodenalis* (syn. *G. lamblia* or *G. intestinalis*, from now on referred to as *Giardia*) is a flagellated protozoan that parasitizes the upper part of the small intestine of mammals, including human, and causes giardiasis, the most common parasitic diarrheal disease worldwide [12]. *Giardia* is a deeply branched eukaryote, closer to fungi and animals than Euglenozoa [13]. The two life stages of *Giardia*, the binucleated trophozoite, which replicates and colonizes the host intestine, and the tetranucleated cyst, the infective and resistant stage which can survive in the external environment, can be reproduced in laboratory. The phylogenetic position of *Giardia* and its minimalistic genomic and cellular organization make this parasite a fascinating model to investigate basic cellular processes and different aspects of eukaryotes' evolution [14].

*Giardia* posseses a single 14-3-3 isoform (g14-3-3) showing high sequence identity to the 14-3-3s of the epsilon subgroup. In previous works we have demonstrated that g14-3-3 is a fully functional member of the family with a central role in multiple biological pathways of *Giardia* [15]–[16]. Interestingly, two post-translational modifications (PTMs) make g14-3-3 unique in the 14-3-3 protein family: constitutive phosphorylation and polyglycylation. More specifically, g14-3-3 is constitutively phosphorylated on residue Thr214, which is located in the loop between α-helices H and I, and it represents the unique known case of 14-3-3 subjected to polyglycylation, which consists in the addition of one or more glycine residues to the γ-carboxyl group of a glutamate [15].

Phosphorylation of different 14-3-3s was previously documented, but it was observed to only occur in certain physiological conditions or cell types and involve residues located either in the dimer interface, thus favoring 14-3-3 monomerization, or in the α-helix G and at the extreme C-terminus, thus modulating the binding to the targets [17]. The phosphorylation of Thr214 may play a role in promoting the binding of g14-3-3 to its targets. In fact, the g14-3-3 phospho-mimicking mutant T214E, expressed as bacterial recombinant protein, displayed an enhanced binding to synthetic phosphopeptides reproducing multiple 14-3-3 binding motifs *in vitro* [18]. Moreover, the expression in *Giardia* of the non phosphorylatable T214A mutant behaved as a dominant negative leading to an impaired cyst development.

As for polyglycylation, this unusual PTM takes place at the penultimate g14-3-3 C-terminal residue, Glu246, and consists in the addition of up to 30 consecutive glycines per monomer. The length of the polyglycine chain is stage-dependent and decreases down to 10 residues during the cyst formation in parallel with a partial re-localization of g14-3-3 to the nuclei [15], [18]. Both the *in vivo* expression of the E246A mutation, which disables the g14-3-3 polyglycylation, and the alteration of the polyglycylation/deglycylation enzyme concentration ratio, affects the intracellular localization of the protein and the parasite development into cyst [18]–[19].

Furthermore, the structural/functional significance of both polyglycylation and phosphorylation has been recently supported by the observation that when the g14-3-3 was expressed in *Drosophila melanogaster* the protein resulted devoid of both PTMs and was unable to complement fly mutants deleted of either the endogenous D14-3-3ε or the DLeoII (a 14-3-3ζ isoform) [20].

Due to the relevance of g14-3-3 in many *Giardia* biological processes (i.e. cyst formation) and the peculiar need for constitutive PTMs for the protein proper activity *in vivo*, structural information is essential to understand how phosphorylation and polyglycylation affect the function of g14-3-3. This understanding would clearly pave the way for the design of antigiardial drugs targeting g14-3-3 binding activity.

In order to better clarify the structural and functional role of g14-3-3 PTMs, we studied the behavior of the protein with and without modifications using three complementary approaches. First, the crystal structure of a g14-3-3 in the apo form is presented and discussed. The structure shows that the protein, in absence of both PTMs, oligomerizes through C-terminal domain swapping events. The formation of filaments was also confirmed by transmission electron microscopy. Second, the combination of various mutants with native PAGE and chemical cross-linking indicates that the oligomerization process does not occur when a polyglycine stretch is added to the protein C-terminus. Finally, molecular dynamic simulations support the hypothesis that the phosphorylation of Thr214 promotes the binding to the target(s).

## Materials and Methods

### Vectors' construction

Escherichia coli JM109 and XL1Blue competent cells were used for vector manipulation and recombinant proteins expression, respectively. Glutathione S-Transferase (GST)-difopein, GST-g14-3-3 (plasmids p14-X) and GST-T214E expression vectors have been described elsewhere [15], [18]. The R200K and the T208A mutants were obtained by site-directed mutagenesis using the p14-X vector15 as template, and the designed primers: R200Kfor 5′-AGGGCTTGCGAGCTTGCGAAGAAGGCTTTCGAC-3′; R200Krev 5′-GGCCGCGTCGAAAGCCTTCTTCGCAAGCTCGCA-3′; T208Afor 5′-GCTTTCGACGCGGCCATCGCAGATCTGGACAAG-3′; T208Arev 5′-CGGTCAGCTTGTCCAGATCTGCGATGGCCGCGTC-3′ (mutated triplets are underlined). The reaction was performed as previously detailed [15] and according to manufacturer's instruction. The obtained plasmids were designed as “pT208A-X” and “pR200K-X”. To obtain the polyG10 and polyG20 mutants in which the last two C-terminal residues were deleted and replaced with a stretch of 10 or 20 glycines, respectively, we took advantage of the presence of a KpnI site at position 404 of g14-3-3 coding sequence and a NotI site in the multicloning site of the pGEX-6P1 vector. A KpnI-NotI cassette was PCR amplified from the p14-X vector15 using the “g14KpnIfor” primer, 5′-CCC*GGTACC*TCGCCGAGTACTCGT-3′ (KpnI restriction site is in italic), in combination with the “polyG10rev”, 5′-GGG*GCGGCCGCTTA*ACCTCCACCTCCTCCACCTCCACCTCCACC**CTCGGCATTATCGTCACC**-3′, or the “polyG20rev” primers, 5′-GGG*GCGGCCGCTTA*ACCTCCACCTCCACCTCCACCTCCACCTCCACCTCCACCTCCTCCACCTCCACCTCCACC**CTCGGCATTATCGTCACC**-3′ (sequence coding for polyglycines stretch is in bold and underlined, the NotI restriction site is in italic and the stop codon is underlined and in italic). The coding sequence of human 14-3-3ζ was PCR amplified from the plasmid pGEX2TK-14-3-3ζ [21] using the primers “14ζforF”, 5′-CCCGGATCCATGGATAAAAATGAGCTGGTTC-3′ (BamHI restriction site is underline), and “14ζrev“, 5′-GGG*GCGGCCGC*TTAATTTTCCCCTCCTTCTCC-3′ (NotI restriction site is in italic). PCR reactions were performed in a final volume of 50 μl using 25 μl of 2X PCR master mix (Promega, France), 20 pmols of each primer and 50 ng of plasmid p14-X [15] as template. Reactions were performed on a T-Personal Thermocycler (Biometra Corporation, Göttingen, Germany). Amplification conditions were: one cycle at 95°C for 5 min; 30 cycles at 95°C for 30 sec, 55°C for 30 sec and 72°C for 30 sec; and one cycle at 72°C for 7 min. The PCR fragments were firstly cloned in the pGEM_Teasy vector (Promega). KpnI/NotI digested polyG10 and polyG20 fragments were sub-cloned in the KpnI/NotI-digested p14-X vector replacing the last 341 nucleotide of the g14-3-3 coding sequence, whereas the BamHI/NotI digested 14-3-3ζ was cloned in the BamHI/NotI-digested pGEX6P1 vector. The obtained plasmids were designated as “pPolyG10-X”, “pPolyG20-X” and “ph14-3-3ζ-X”, respectively.

### Proteins expression and purification

For protein expression, transformed *E. coli* were grown in SOB medium at OD_600_ = 0.6–0.8, and expression induced with 0.5 mM isopropyl thio-β-D-galactoside (IPTG) at 37°C for 4 h. GST-fused proteins were purified by affinity chromatography on glutathione-sepharose 4B (GE Healthcare, Uppsala, Sweden) and released from GST by digestion with PreScission protease (GE Healthcare) at 4°C for 16 h in digestion buffer according to manufacturer. Protein were diafiltrated o.n. at 4°C either in 50 mM Tris-HCl pH 7.5 (for crystallization) or in 10 mM Tris-HCl pH 7.0, for Circular Dicroism (CD) experiments, using a PM-5 membrane. Proteins were concentrated using Centricon 10 (Millipore Corporation, Bedford, MA, USA) and concentration was measured with Bradford's method (BioRad, Hercules, CA, USA).

### Circular dicroism

CD spectra were measured with a Jasco J-715 spectropolarimeter (Jasco Ltd, Hachioji City, Tokyo, Japan) in 1.0 cm quartz cuvettes between 260 and 195 nm. Samples were 0.1 μg/μl in 10 mM Tris/HCl buffer (pH 7.0).

### Protein crystallization, data collection and processing

Crystals were grown by the hanging drop vapor diffusion method at 293 K. Protein sample was concentrated to 10.5 mg/ml. Crystallization drops consisted of 1 μl of protein solution mixed with an equal volume of the reservoir solution on a cover slip which was suspended over the reservoir containing 2 M ammonium sulfate, 5% (w/v) PEG400 and 0.1 M HEPES pH 7.5. Crystals, grown in 1–2 weeks, were cryo-protected with 20% glycerol, mounted in nylon loops and flash-cooled by submersion into liquid N_2_ for transport to the synchrotron-radiation source. Crystals are poorly reproducible and most of them diffract at low resolution. X-ray diffraction data were collected at 3.2 Å resolution as 0.5° oscillation frames at 100 K on the beam line ID14-1 at ESRF (Grenoble, France) using a CCD detector. The data were processed using the HKL2000 package [22] and the CCP4 suite [23]. The structure was solved by molecular replacement with the program Phaser [24] using the structure of human 14-3-3ε as search model (PDB code 2BR9) [25] which displays 63% sequence identity with g14-3-3. Manual fitting and model building were performed using COOT [26]. Refinement was performed using Refmac5 [27]. Structural figures were generated with PyMol [28]. Crystal parameters, data-collection and refinement statistics are presented in Table 1. Atomic coordinates and structure factors have been deposited in the Protein Data Bank with accession number 4F7R. Protein Interfaces, Surfaces and Assemblies (PISA) Service [29] at the European Bioinformatics Institute (http://www.ebi.ac.uk/msd-srv/prot_int/pistart.html) was used for protein-protein complex analyses. The structural alignment between proteins was performed using the software Superpose (Secondary Structure Matching) [30].

**Table 1:** Crystal parameters, data collection statistics and refinement statistics of g14-3-3.

PDB ID	4F7R
Space group	P3_2_
Unit cell parameters (Å)	
*a*	100.9
*c*	140.5
No. of molecules in the asymmetric unit	4
<*B*> for atomic model (Å^2^)	54.5
Resolution ranges (Å)	50.0–3.2 (3.31–3.20)
Unique reflections	26426
Completeness (%)	100 (100)
Redundancy	7.0 (7.0)
*R*_merge_ a	12.5 (76.2)
? ^2^ b	1.06 (1.2)
<*I*/σ(*I*)>	16.3 (2.8)
*R*_crys_ (%)	18.5 (29.4)
*R*_free_ (%)	23.1 (31.9)
rms (angles) (°)	0.812
rms (bonds) (Å)	0.005
Residues in core region of Ramachandran plot (%)	930 (98.9)
Residues in generously allowed region of Ramachandran plot (%)	10 (1.1)
Residues in disallowed region of Ramachandran plot (%)	0

Values in parentheses are for the highest-resolution shell.

a *R*_merge_  =  ∑*_hkl_*∑*_i_*|*I_i_*(*hkl*) − <*I*(*hkl*)>|/∑*_hkl_*∑*_i_I_i_*(*hkl*), where *I_i_*(*hkl*) is the *i*th observation of the reflection (*hkl*) and <*I*(*hkl*)> is the mean intensity of the (*hkl*) reflection.

b χ^2^  =  ∑*_ii_*(|*I_ij_*(*hkl*) − <*I_i_*(*hkl*)>|)^2^/(σ*_i_* ^2^ *N*/(*N* −1).

### Molecular dynamic simulations

The *in silico* phosphorylation of Thr214 was modeled on the three-dimensional crystal structure of g14-3-3 (PDB:4F7R) using the psfgen module of NAMD [31]. The molecular dynamics simulation was performed using the NAMD 2.8 package for GPU computing [31]. The g14-3-3 structure was immersed in a rectangular simulative box filled with 45.000 TIP3P [32] explicit solvent molecules and rendered electro neutral by the introduction of 4 sodium counter-ions. A standard CHARMM force field [33] was adopted to parameterize the system for bond parameters and Lennard-Jones terms [34]. The length of hydrogen bonds was constraint by applying the LINCS algorithm [35] and the electrostatic interactions were taken into account by means of the Particle Mesh Ewald method [36]. A first round of 5000-step minimization was performed in order to regularize the structure of all the atoms composing the system, using the Steepest Descent algorithm [37]. Optimization and relaxation of solvent and ions was performed by simulating the system at a temperature of 50, 100, 150, 200, 250 K, for 500 ps each with a time step of 1.0 fs. Simulations were performed at a constant temperature of 300 K using the Langevin method and at constant pressure; the pressure was kept constant (1 bar) using the Rahman-Parrinello barostat [38] and 2.0 fs time step. Principal component analysis (PCA), structural clustering, and analyses of RMSD, RMSF, solvent accessible surface area, and salt bridges were carried out using the GROMACS 4.5.4 analysis tools [39]. PCA (i.e. Essential Dynamics) was performed as previously described [40]. Briefly, higher frequency fluctuations were filtered out by diagonalizing the atomic positional fluctuation covariance matrix calculated during the production run. The corresponding eigenvectors and eigenvalues were used to describe large-amplitude motions. The first few eigenvectors of the diagonalized covariance matrix usually account for the major fraction of the total variance. The projection of the atomic trajectories onto the corresponding eigenvectors represents large collective atomic motions. The images were obtained with PyMol and UCSF Chimera [41] and the graphs were calculated with XMGRACE (Paul J. Turner Center for Coastal and Land-Margin Research Oregon Graduate Institute of Science and Technology Beaverton, Oregon).

### Purification of endogenous g14-3-3

Trophozoite of *Giardia duodenalis* WBC6 strain were grown as previously described [15]. Parasite's protein lysate was prepared as previously described [18] and endogenous g14-3-3 was affinity purified on glutathione-Sepharose immobilized GST-difopein and g14-3-3 eluited with 100 μl of 5 mM “Raf1p” phosphopeptide, (LSQRQRST(pS)TPNVHMV), reproducing one of the 14-3-3-binding motifs of the human Raf-1 protein [15]. The excess of phosphopeptide was removed by dialysis against HT buffer (25 mM Hepes-KOH pH 7.6, 75 mM KCl, 5 mM MgCl_2_, 0.1 mM EDTA, 0.04% Tween 20) with Centricon 10 (Millipore Corporation, Bedford, MA, USA).

### Mass spectrometry analysis

Affinity purified g14-3-3 was separated on 1D-gel NuPAGE 4–12% (Novex, Invitrogen) run in morpholinepropanesulfoninic acid (MOPS) buffer and stained with the Colloidal Blue Staining kit (Invitrogen). For the MS/MS identification of phosphorylated residue in g1433, in gel digestion and nanoflow reversed-phase liquid chromatography tandem mass spectrometry (RP-LC-MS/MS) analysis were performed, with minimal changes, as previously described [16] using an HPLC Ultimate 3000 (DIONEX, Sunnyvale, CA U.S.A) on line connected with a linear Ion Trap (LTQ-XL, ThermoElectron, San Jose, CA). MS data were searched for matching with the *Giardia* protein database, downloaded from the web site http://www.giardiadb.org/giardiadb, considering peptides specifically cleaved by trypsin on K or R, possibly modified by oxidation on methionine and/or phosphorylation on S, T or Y residues (Δm: + 80 Da).

### Cross-linking Studies and native page

For cross-linking studies, recombinant proteins were dialyzed in HT buffer with Centricon 10 (Millipore Corporation, Bedford, MA, USA). A final concentration of 0.33 μM recombinant proteins, or affinity purified endogenous g14-3-3, were pre-incubate with “Raf1p” phosphopeptide (0.66 μM) in 5 μl of HT buffer for 16 h at 4°C either in presence or absence of 1 mM DTT and then subjected to cross-linking by the addition of 5 μl of 0,4 M triethanolammine pH 8.0 with or without 300 μM DMP (dimethyl pimelimidate) for 5 min at 37°C. Reaction was stopped with 5 μl of 150 mM Tris-HCl pH 8.0. Samples were mixed with 5 μl of 4X loading buffer (Novex, Invitrogen) and 2 μl of sample reducing agent (Novex, Invitrogen), separated on NuPAGE 4–12% gel (Novex, Invitrogen) in morpholinepropanesulfonic acid (MOPS) buffer, transferred on nitrocellulose (BioRad, Hercules, CA, USA) and probed with the rabbit anti-g14-3-3 serum (serum N14 at 1∶5000) [15] or the rabbit anti-human 14-3-3 antibody Ab14112 (at 1∶500, Abcam plc, Cambridge, UK). Antibody/antigen interaction was revealed by incubation with anti-rabbit HRP-conjugated secondary antibody (Ab) (1∶2,000) and chemioluminescence (Millipore Corporation, Bedford, MA, USA). For native PAGE, 5 μM of recombinant proteins or affinity purified endogenous g14-3-3 were incubated in HT buffer with 10 μM of “Raf1p” peptide or the “Raf1” unphosphorylated one 16 h at 4°C. Native PAGE was performed on 12% PAGE as previously described [42] and gel stained with 0.25% (w/v) Coomassie Brilliant Blue R-250.

### Transmission Electron Microscopy (TEM)

Recombinant Prescission-cleaved g14-3-3 and polyG20 mutant were incubated at 4°C at 0.1 or 1 μg/μl in 25 mM Hepes-KOH pH 7.6 for 16 h. Aliquots of 10 μl of protein solutions (0.1 μg/μl and 1 μg/μl) were applied onto formvar-carbon coated copper grids. After 5 minutes for adsorption, the excess of protein solution was sucked off. The air-dried grids were stained with an aqueous solution of 1% (w/v) uranyl acetate for 15 s. The excess of staining solution was removed carefully. The grids were observed with a PHILIPS EM208S transmission electron microscope at an acceleration voltage of 80 kV. Electron micrographs were taken with a slow scan camera (Megaview III, Olympus).

### Accession number

The PDB accession number for the g14-3-3 reported in this paper is 4F7R.

## Results

### The overall structure of the g14-3-3

To achieve an amount of g14-3-3 suitable for structural analyses we used an N-terminally GST-fused g14-3-3 expressed in bacteria that was characterized in previous works for its ability to bind both to several phosphopeptides reproducing 14-3-3 binding motifs and to the phosphorylation-independent binding peptide difopein [15], [18]. Although the g14-3-3-specific polyglycylase gTTLL3 (Tubulin Tyrosin Ligase-Like3) had already been identified and expressed [19], the low efficiency of the *in vitro* polyglycylation process did not allow us to to obtain sufficient polyglycylated protein for crystallization studies. On the other hand, the g14-3-3-specific protein kinase is still unknown. Notice that the GST-cleaved full-length unmodified g14-3-3 used for the crystallization experiments only differed from the native g14-3-3 for the presence of a GPLGS sequence upstream the initial methionine.

**Figure 1:**
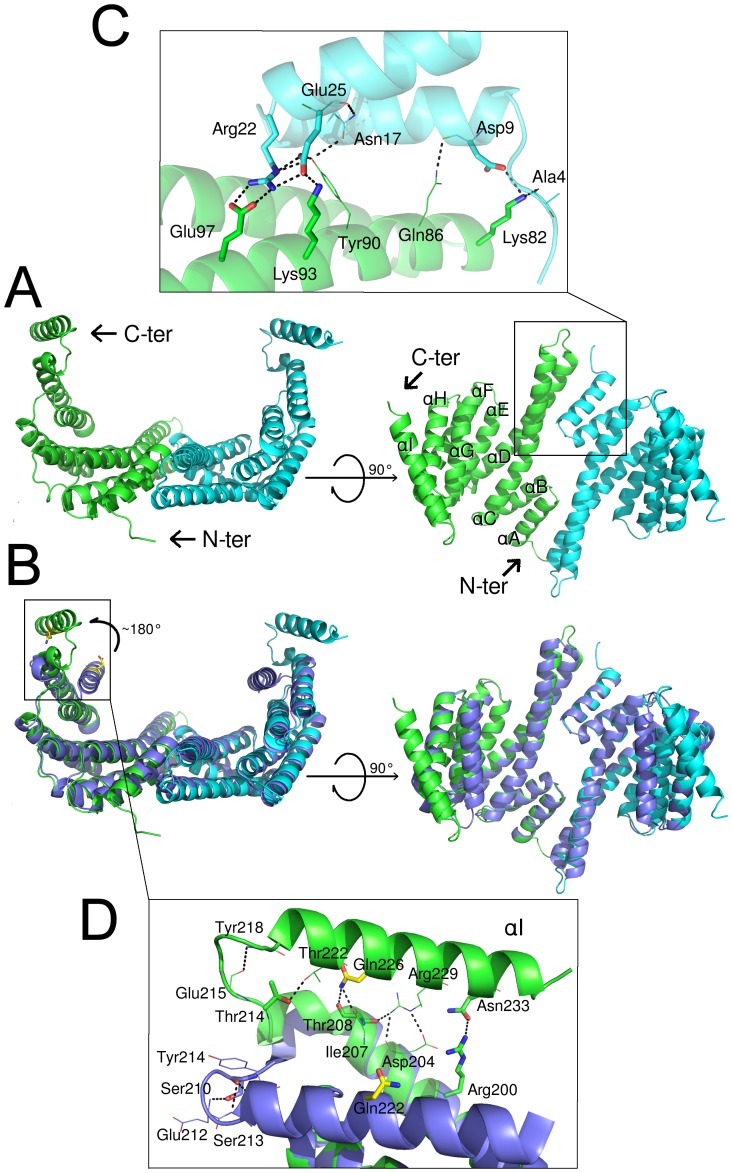
Classical dimer of g14-3-3 assumes an “open” conformation. A) Two views of g14-3-3 N-terminal dimer (green, cyan). B) The superimposition of g14-3-3 and human 14-3-3ε (2BR9, violet) emphasizes the open conformation of g14-3-3. C) Close up of N-terminal dimerization interface. Residues are shown as lines if involved in hydrogen bonds, as sticks if involved in salt bridges. D) Detail of polar interactions between helices H and I. Residues not conserved in 14-3-3 family are represented as sticks. A conserved residue (Gln226 in g14-3-3 Gln222 in human 14-3-3ε) is shown as yellow sticks in B) and D) to highlight the different orientations of helix I.

**Figure 2:**
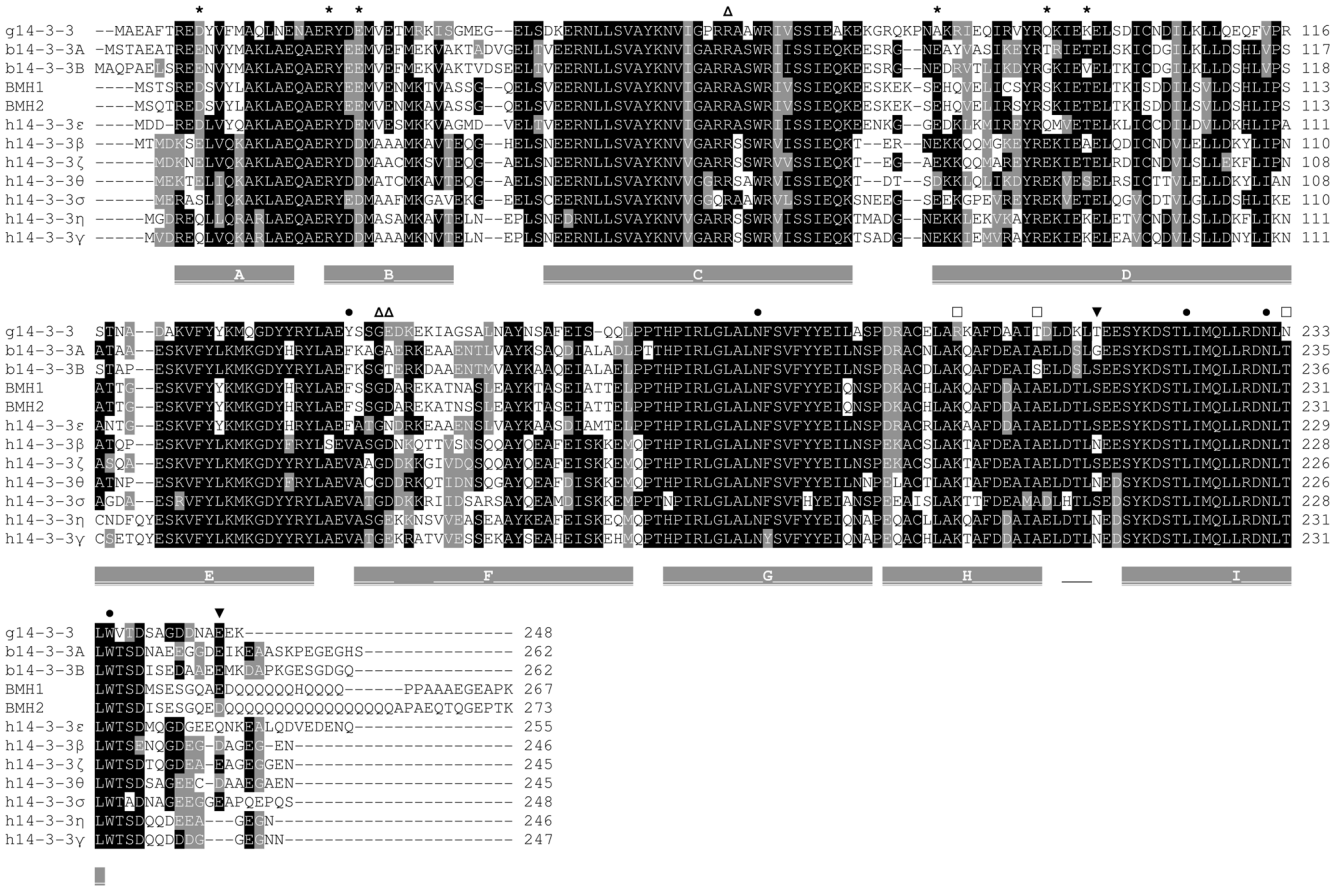
Sequence alignment of g14-3-3 protein. The g14-3-3 protein has been aligned with two *Ordeum vulgaris* isoforms, one from epsilon and one from non-epsilon plant 14-3-3 subgroups (b14-3-3A, P29305.1; b14-3-3B, Q43470.1, respectively), the two *Saccharomyces cerevisiae* yeast isoforms (BMH1, P29311.4; BMH2, P34730.3) and the seven human 14-3-3 isoforms (h14-3-3β, accession number NP_647539.1; h14-3-3τ, NP_006817.1; h14-3-3η, NP_003396.1; h14-3-3ζ, NP_003397.1; h14-3-3γ, NP_036611.2; h14-3-3σ, NP_006133.1; h14-3-3ε, NP_006752.1). Alignment has been generated using ClustalW2 software and edited with BOXSHADE 3.21. Identical residues are black boxed, similar residues are gray boxed, divergent ones are left unboxed. Dashes indicate gaps. A grey line above the alignment indicates the α- helices, the thin line indicates a region of helix3_10_, observed also in h14-3-3 γ, ε, η and σ. Residues involved in N-terminal dimerization are indicated by stars; the triad of residues contacting the phosphate moiety in the target phosphopeptide are indicated by empty triangles. Dots indicate other residues taking contact with the target phosphopeptide backbone and lateral chains. Black triangles indicate Thr214 and Glu246 of g14-3-3. White squares indicate the residues Arg200, Thr208, and Asn233 forming intermolecular hydrogen bound in the C-terminal dimer interface.

The structure of the g14-3-3 was determined at 3.2 Å resolution in the P3_2_ space group and the asymmetric unit contained two functional dimers (Fig. 1A–1B). Each g14-3-3 monomer displayed the typical 14-3-3 structural organization [5]–[7] with nine antiparallel α-helices (A-I) arranged in a ‘U’ shaped fashion with a large central binding groove. Each monomer could be divided into two sub-domains: the N-terminal sub-domain (helices A-D), which consisted of the canonical dimerization interface as well as the floor of the binding groove, and the C-terminal sub-domain (helices E-H), which comprised the side walls and the roof of the binding groove (Fig. 1A-1B). The g14-3-3 buried N-terminal dimerization interface was 1240 Å^2^ per monomer (8.5% of the total accessible surface area), scored 0.3 in Complexation Significance Score (CSS), indicating that the dimerization was not due to crystal packing and confirming, as expected, the relevance of the g14-3-3 dimerization surface. The g14-3-3 N-terminal dimerization relies on residues largely conserved among the 14-3-3 family members [6], [25] involving, in this case, three salt bridges between Arg22 and Glu97, Asp9 and Lys82, and Glu25 and Lys93 (Fig. 1A and 1C). Arg22, Asp9, and Glu25 are located in g14-3-3 α-helices A and B of one monomer whereas Glu97, Lys82, Lys93 reside in α-helix D of the other protomer. In the homodimeric human 14-3-3ε, as well as in the epsilon-like Cp14e from the protozoan parasite *Cryptosporidium parvum*, dimerization involves only the first salt bridge (Arg19-Glu92 in h14-3-3ε and Arg43-Glu119 in epsilon-like Cp14e, respectively) [7], [25]. The presence of a single salt bridge allows h14-3-3ε to mainly heterodimerize with other human isoforms [25]. Interestingly, g14-3-3 dimerized through three salt bridges similar to 14-3-3ζ [4]-[41] despite the higher sequence identity of g14-3-3 with proteins of the 14-3-3ε subgroup than with those of the 14-3-3ζ subgroup. This suggests that g14-3-3 only forms stable homodimers. In support of this, when g14-3-3 was expressed in *Drosophyla melanogaster* or when fly isoforms were expressed in *Giardia* heterodimerization occurred in both cases only between g14-3-3 and D14-3-3ζ (LeoII) but never between g14-3-3 and D14-3-3ε [20]. The three-dimensional superimposition of the g14-3-3 and h14-3-3ε structures yielded a root mean square deviation (rmsd) of 0.95 Å. This low rmsd value indicates that helices A-H are largely super-imposable. The software automatically excluded helix I from the calculation as it was recognized as a largely dissimilar region. Indeed, helix I of g14-3-3, compared with the human isoforms, is rotated of about 180° around helix H (Fig. 1B and 1D) implying a substantial rearrangement of the loop connecting helices H and I (HI loop). The displacement of the HI loop causes the disruption of a “ST-motif” found in most 14-3-3s and including Ser210 (according to h14-3-3ζ numbering), which corresponds to Thr214 in g14-3-3. (Fig. 1D). This motif consists of 4–5 residues including a Ser or a Thr forming hydrogen bonds with two residues downstream in the sequence [43]. Instead, the displacement of the HI loop in g14-3-3 is favored by eight hydrogen bonds between helices H and I (Fig. 1D) that involve, among others, residues Arg200, Thr208, Thr214 and Asn233 which correspond, in the human isoforms, to Lys194, Ala202, Ser/Asn208 and Thr227, respectively (according to h14-3-3ζ numbering) (Fig. 2).

As consequence of the helix I rearrangement, the g14-3-3 structure showed a fully open “extended” conformation of the C-terminal region, supporting the notion that the g14-3-3 region encompassing helices G and I possesses a degree of flexibility allowing the binding groove to change from a “closed” to an “open” conformation and thus accommodating different types of peptides [25].

### C-terminal dimer-dimer interface

**Figure 3:**
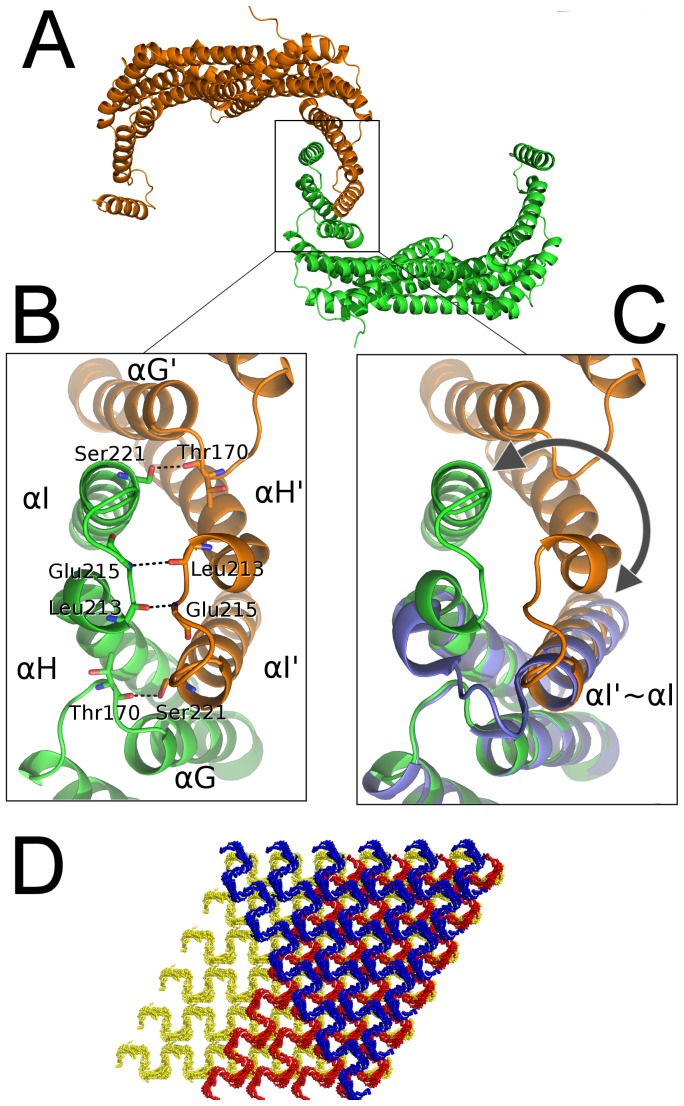
C-terminal domain swapping leads to fibrils formation in the crystal. A) Two adjacent dimers within the crystal (green, orange), corresponding to one asymmetric unit, give rise to non-canonical C-terminal dimerization through the swapping of helix I. B) Detail of the interface showing the residues involved in hydrogen bonds. C) The superimposition of g14-3-3 and human 14-3-3ε (violet) emphasizes the swapping of helix I (g14-3-3 αI' vs h14-3-3ε αI) as indicated by the arrow. D) Crystal packing (36 unit cells, 6x6x1). The crystal is formed by layers (blue, red, yellow) of endless parallel filaments.

The described g14-3-3 “extended” conformation entails the exposure of several hydrophobic residues located in helix G, H and I and usually buried in other 14-3-3s. A possible consequence of such exposure of hydrophobic residues is the observed domain swapping occurring between the C-terminus of two g14-3-3 monomers, each belonging to a different dimer, in which helix I' from a monomer interacts with helices F and H from the monomer of the neighboring dimer (Fig. 3A; B and C). In contrast, in other available structures of 14-3-3 dimers, helix I is observed to interact with helices F and H from the same monomer [6]. Domain swapping entails a dimerization event at the protein C-terminus with the formation of a four-helix bundle between two adjacent g14-3-3 dimers (Fig. 3B and 3C), leading to the assemblage of protein filaments. Domain swapping was observed in the structure of different proteins and it is defined as two identical protein chains exchanging a part of their structure to form an intertwined dimer or a higher-order oligomer [44]. The overall g14-3-3 crystal packing resulted in stacks of endless parallel filaments (Fig. 3D), each layer being rotated 60° with respect to the previous one. The calculated C-terminal dimer-dimer buried interface is 1576.9 Å^2^ per monomer, i.e. about 11% of the total accessible surface area. The calculated solvation free energy gained upon formation of the interface (ΔG  =  -39.3 kcal/mol) suggests that the oligomer is highly stable. As previously stated, a stretch of hydrophobic residues is exposed at the C-terminus of each monomer so that the C-terminal dimer-dimer interface results predominantly hydrophobic and further stabilized by four intermolecular hydrogen bonds between Ser221 of helix I and Thr170 of loop FG' (and Ser221 of helix I' and Thr170 of loop FG), and Glu215 of loop HI and Leu213 of helix H' (and Glu215 of loop HI' and Leu213 of helix H). Nearly, all the described intermolecular interactions in g14-3-3 can be also observed in the available 14-3-3 structures in the form of intra-molecular interactions involving helix I, which perfectly replaces the swapped helix I' (Fig. 3B). The only exception is represented by the hydrogen bond involving loop HI (residues Glu215-Leu213) that does not occur in the non-swapped dimer (Fig. 3B).

### The g14-3-3 target binding site

The 14-3-3 binding to its target(s) primarily occurs through the interaction of conserved residues in the 14-3-3 binding groove and a phosphorylated peptide in the target(s). According to h14-3-3ε numbering, the pSer/pThr phosphate moiety of the ligand binds to a highly conserved 14-3-3 triad formed by Arg57, Arg130 and Tyr131 whilst Asn176 and Asn227 interact with the phosphopeptide backbone [6], [25].

**Figure 4:**
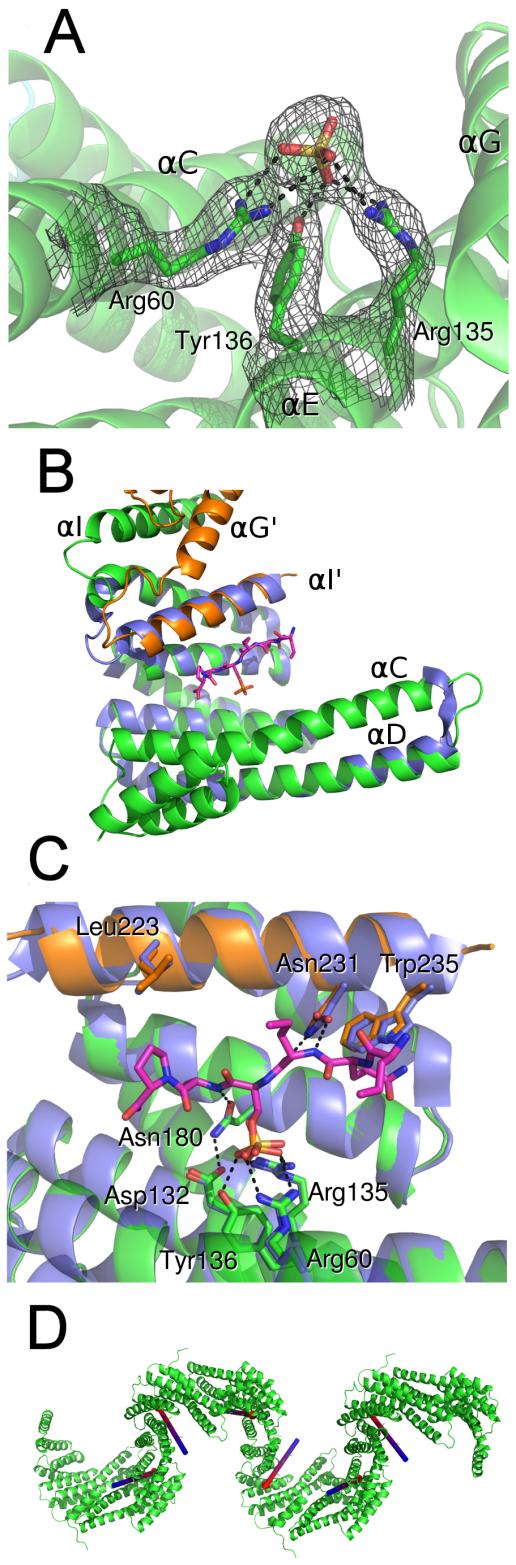
Phosphopeptide binding site is preserved upon helix I swapping. A) A sulfate ion bound in correspondence of the phosphate binding site in g14-3-3. 2mFo-dFc electron density (contoured at 1σ) is shown for sulfate ion and the residues involved in the interaction. B) Superimposition of g14-3-3 (green, orange) and 14-3-3ε-phosphopeptide (2BR9, violet-pink) complex showing that the swapped helix I' can participate to peptide binding. C) Detail of the peptide-binding site. The peptide (from the 2BR9 structure) and the residues involved in the interaction, all conserved in g14-3-3, are represented as sticks. D) Orientation of the phosphopeptides modeled into a g14-3-3 filament. The phosphopeptides in the binding cleft are represented by arrows blue to red(N-terminus to C-terminus).

In the g14-3-3 structure, despite helix I swapping, the conformation of the residues involved in the recognition and binding to phosphopeptide(s) resulted to be conserved (Fig. 4). Indeed, the visual inspection of the Fo-Fc electron density map revealed a peak that was assigned to a sulphate ion (Sup. Fig. S1), present in the crystallization solution, which lies in the phosphate binding site and interacts with the g14-3-3 Arg60-Arg135-Tyr136 triad (Fig. 4A and Sup. Fig. S1), thus mimicking the phosphate moiety of a pSer/pThr binding peptide. This was supported by the superimposition between g14-3-3 (in the apo form) and the human 14-3-3ε/RRQR(pS)AP phosphopeptide complex (PDB 2BR9). All the residues involved in the phosphopeptide binding (Arg60, Arg135, Asp132, Tyr136, Asn180, Asn231, according to g14-3-3 numbering) are conserved, although it is worth noting that Asn231 belongs to helix I' in g14-3-3 (Fig. 4B-4C). The observed “extended” conformation of g14-3-3 with the large conformational change of helix I would disrupt the binding groove, which is indeed restored by domain swapping of helix I', indicating that the protein assemblies can retain phosphopeptide binding properties even in the case of C-terminal dimerization (Fig. 4B–D).

### Structural role of g14-3-3 phosphorylation

**Figure 5:**
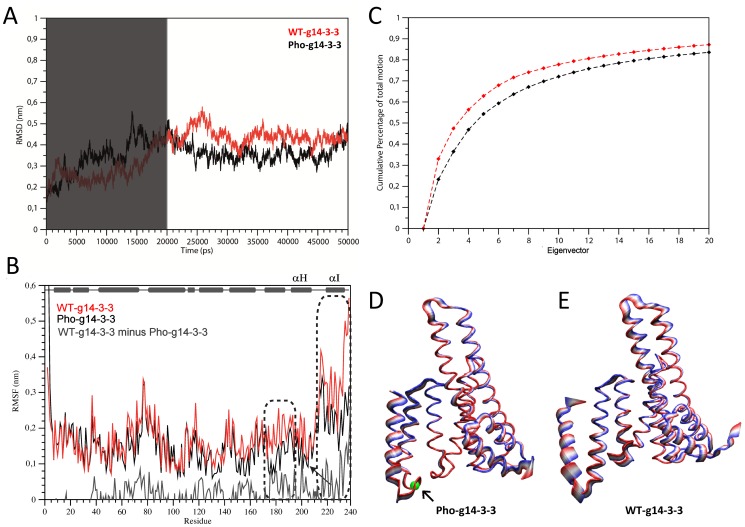
Molecular Dynamic simulation. A) The evolution of the RMSD (Root Mean Square Deviation) of WT-g14-3-3 (in red) and Pho-g14-3-3 (in black) during the simulation. In order to allow the system to equilibrate, the first 20 ns of simulation were discarded from the analyses (region shaded in grey). B) The per residue RMSF (Root Mean Square Fluctuation). Helices H and I are enclosed in dashed boxes. The position of Thr214 is indicated with an arrow. The bottom plot (grey) shows the per residue RMSF difference between WT-g14-3-3 and Pho-g14-3-3. A schematic secondary structure diagram of the g14-3-3 is reported on the top. C) Eigenvectors cumulative weight on total motion for WT-g14-3-3 (in red) and Pho-g14-3-3 (in black). Projection of motion along the first eigenvector for Pho-g14-3-3 D) and the WT-g14-3-3 E) protein structures. The amplitude of motion follows the color scale from red to blue. The phosphorylated residue is represented in grey and indicated with an arrow.

**Figure 6:**
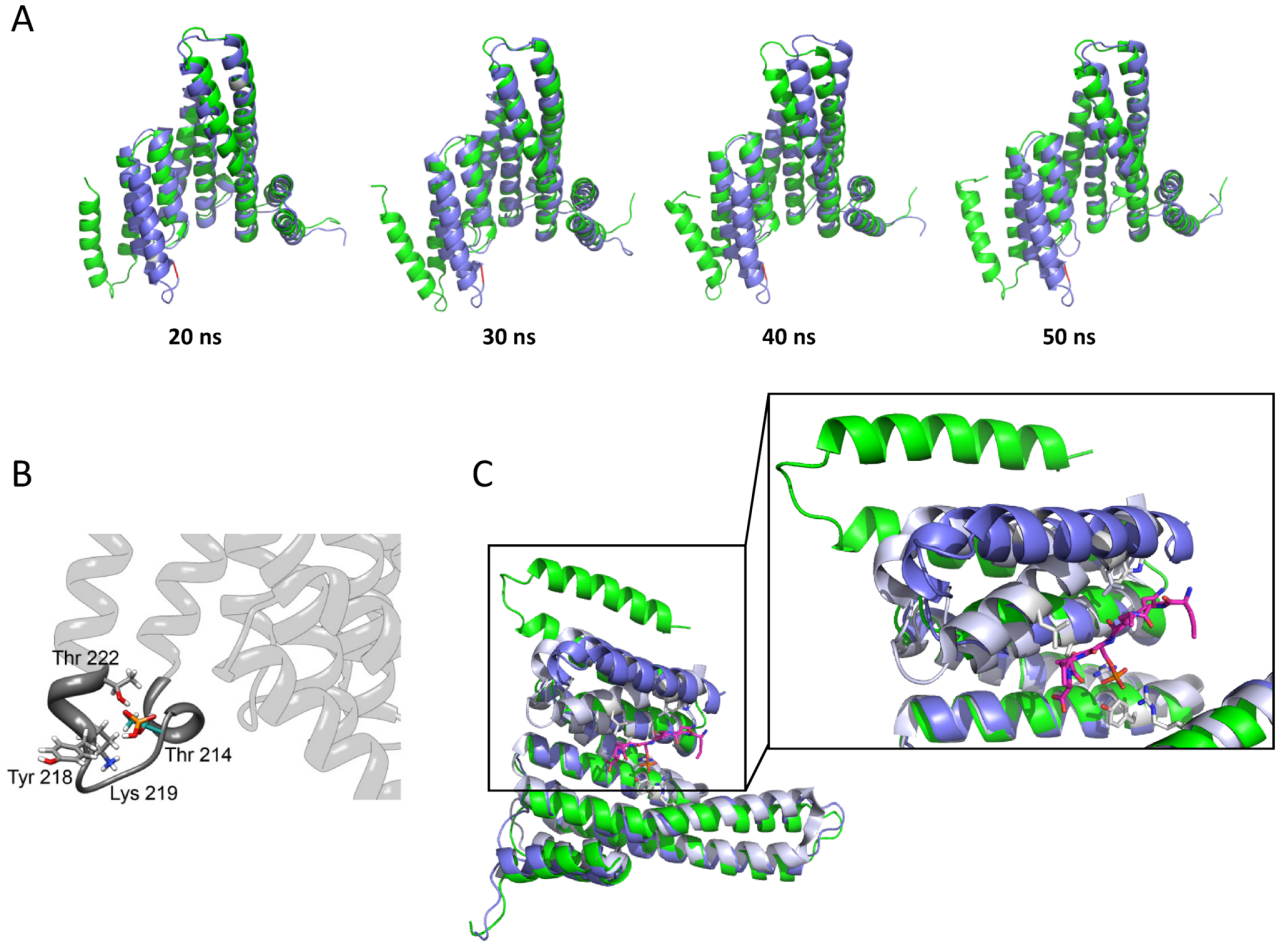
Molecular Dynamic simulation. A) Snapshots at 20, 30, 40, and 50 ns of the protein structures during the simulation. The proteins are superimposed to each other, WT-g14-3-3 is in green and Pho-g14-3-3 is in violet. B) Magnification of the loop between helices H and I. The phosphorylated Thr214 and the surrounding polar residues are represented in sticks colored by atom. C) Superimposition Pho-g14-3-3 at 20 ns of MD (violet), WT-g14-3-3 (green) and h14-3-3ε (grey) in complex with phosphopeptide (represented as sticks in pink-blue) showing that upon phosphorylation g14-3-3 helix I repositions closest to helix H but still doesn't overlap with h14-3-3ε helix I. The h14-3-3ε residues involved in the interaction with the peptide are represented as stick (grey).

The solved structure of g14-3-3 is not phosphorylated and Thr214 is buried at the C-terminal interface (Fig. 1D) and its side chain establishes a hydrogen bond with Thr222. Therefore, it seems likely that phosphorylation alters the Thr214 pattern of interactions (i.e. potentially affecting the local protein folding or energy) thus influencing target binding and/or domain swapping. In order to better understand how phosphorylation of Thr214 may affect the protein motion, the effects of phosphorylation on the dynamical behavior of g14-3-3 were analyzed by *in silico* phosphorylation of Thr214 in the 3D structure of the g14-3-3 monomer and MD simulations on both the unmodified (WT-g14-3-3) and the phosphorylated systems (Pho-g14-3-3). An MD simulation of 50 ns was run and the first 20 ns were discarded to take into account the system equilibration so that all the analyses were carried out on the last 30 ns (Fig. 5A). The effects of the phosphate group on the protein motion are clearly visible (Fig. 5B). In the WT-g14-3-3 monomer, helices H and I fluctuated much more than in Pho-g14-3-3. The calculated per residue Root Mean Square Fluctuation (RMSF) showed that residues 1-170 fluctuated similarly in WT-g14-3-3 and Pho-g14-3-3, whereas differences could be observed in the helices upstream and downstream Thr214 (Fig. 5B). Interestingly, fluctuations of the loop HI linking helices H and I were similar in the two systems. The behavior of the C-terminal of the protein upon phosphorylation was even more evident in the principal component analysis (PCA) results (Fig. 5C, 5D and 5E). The first 10 eigenvectors (Fig. 5B) described about the 70% of the fluctuations in the essential space and represented most of the protein motion in both phosphorylated and non-phosphorylated proteins (each dot in the plot represents an eigenvector). However, the weight of the first two eigenvector represents about the 35% of the global fluctuations in the WT-g14-3-3 monomer (Fig. 5C), and only the 25% in Pho-g14-3-3 (Fig. 5C). The first two eigenvectors described predominantly the motion of helices H and I, which were subjected to larger movements in WT-g14-3-3 (Fig. 5E) than in Pho-g14-3-3 (Fig.5D), where they were also closer to each other. The results of the PCA were consistent with the MD snapshots taken at 20, 30, 40, and 50 ns (Fig. 6A). Overall, the computational study showed that the phosphorylated residue acts as a “lock” in the elbow between helix H and I. The negatively charged phosphate, indeed, could still allow the interaction with the polar side chain of Thr222, as the non-phosphorylated Thr214 did, and additionally formed a network of interactions with the polar side chain of Tyr218 and with the positively charged side chain of Lys219 (Fig. 6B), by establishing several hydrogen bonds and a salt bridge. The stiffening of the hinge between helices H and I in presence of the phosphate group forced the two helices to keep a much closed conformation during the simulation and oscillated much less than in the non-phosphorylated protein. These interactions were much weaker, or even missed, in WT-g14-3-3 where the negatively charged phosphate group on Thr214 is absent. The lack of such interaction network in WT-g14-3-3 made helix I more flexible and capable of moving away from helix H, thus permitting the observed “extended” conformation of the protein C-terminal. Despite the more closed conformation imposed by the phosphorylation of Thr214, the structural superimposition of Pho-g14-3-3 (at 20 ns of MD), WT-g14-3-3 and the h14-3-3ε/RRQRpSAP complex (Fig. 6C) shows that the Pho-g14-3-3 helix I does not overlap with helix I of h14-3-3ε, as indeed the swapped helix I' of g14-3-3 does (Fig. 4), leading to the possibility that phosphorylation alone might not be sufficient to both hide the patch of hydrophobic residues that are in the C-terminal dimer interface and impair the C-terminal domain swapping.

### Effect of amino acid substitutions and PTMs on oligomerization

**Figure 7:**
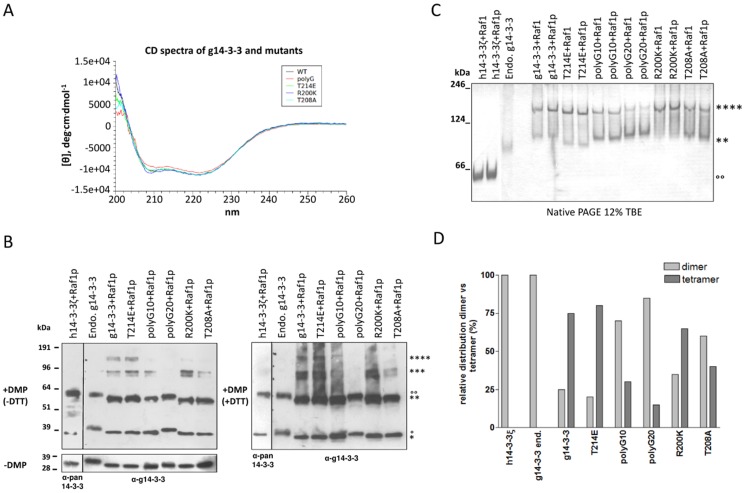
Assessment of g14-3-3 multimerization in vitro. A) Far-UV CD spectra of 14-3-3 proteins. WT (black), T214E mutant (green), T208A mutant (cyan), R200K mutant (violet), polyglycinated 14-3-3 (red). CD spectra were measured with a Jasco J-715 spectropolarimeter (Jasco Ltd, Hachioji City, Tokyo, Japan) in 1.0 cm quartz cuvettes between 260 and 195 nm. Samples were 0.1 μg/μl in 10 mM Tris/HCl buffer (pH 7.0). B) Cross-linking assay. Proteins, pre-incubated with Raf1p phosphopeptide, were incubated with DMP (upper panels), in presence or absence of DTT, or without DMP (lower panel), separated on 4–12% reducing SDS-PAGE and immunoblotted with the indicated antibodies. Asterisks indicate: protein monomer (*), dimer (**), trimer (***) and tetramer (****). Molecular size markers (kDa) are on the left. C) Coomassie staining of 12% basic native PAGE. Proteins were either pre-incubated with Raf1p phosphopeptide or the unphosphorylated peptide Raf1. NativeMark (Invitrogen) size markers (kDa) are on the left. Asterisks indicate g14-3-3 and mutants: dimer (**) and tetramer (****). Empty dots indicate h14-3-3ζ dimer (°°). It is to note that, despite the theoretical molecular weight of g14-3-3 and h14-3-3ζ are comparable (28.9 and 28.1 kDa, respectively) their theoretical isoelectric point (Ip) are much divergent (5.09 and 4.73, respectively) thus contributing to the faster migration of h14-3-3ζ homodimer in native PAGE. Similarly, the presence of either phosphorylated T214 in the endogenous g14-3-3 or the glutamic acid at the same position in the T214E mutant decrease the theoretical Ip from 5.09 to 5.03 and 5.01, respectively, thus accounting for the increased migration of the proteins in the gel. D) Densitometric analysis of the native PAGE presented in (C) performed with ImageJ software. In the graphs: the optic density (OD) of each protein band for each lane is reported as relative percentage (%) of the total of optic density (OD) per lane and expressed as relative distribution of g14-3-3 dimer vs tetramer.

In order to confirm the tendency of recombinant g14-3-3 to form multimers and shed light on the mechanisms leading to the observed “extended” conformation and domain swapping, different g14-3-3 mutants were constructed. Mutants were analyzed by native PAGE and chemical cross-linking, two well established methods already used to asses h14-3-3ζ dimerization properties [42]. As previously observed, residues Arg200, Thr208, and Asn233, which are unique to g14-3-3, may contribute to promote/stabilize the “extended” conformation and the co-occurring C-terminal domain swapping. For this reason, two of them were mutated, Arg200 into lysine (R200K) and Thr208 into alanine (T208A). Lysine and alanine are the residues found in the corresponding positions in all human 14-3-3 isoforms (Fig. 2). Furthermore, due to the relevance of polyglycylation, two mutants were constructed in which the last two C-terminal residues of g14-3-3 were replaced by a stretch of 10 or 20 glycines (polyG10 and polyG20). These mutants could potentially mimic both the presence and the variation in length of the polyglycine chain occurring during *Giardia* encystation [15]. In addition, the previously described T214E mutant was also included [18]. As controls, the well-characterized h14-3-3ζ and the affinity-purified endogenous g14-3-3 from *Giardia* trophozoites, which are physiologically phosphorylated at Thr214 (Sup. Fig. S2), and with different length of polyglycine chain at Glu246 [15], were also analyzed. No variation in the secondary structure could be evidenced by circular dicroism analyses between the unmodified recombinant g14-3-3 and the mutant proteins (Fig. 7A).

As a result of the chemical cross-linking reactions using DMP (9.2 Å spacer arm), bands compatible with the formation of covalent dimer products were visible for both the endogenous g14-3-3 and, as expected, for the h14-3-3ζ [42], as consequence of the N-terminal dimerization (Fig. 7B, left panel). In agreement with the solved crystal structure, dimeric, trimeric and tetrameric forms were clearly visible in the recombinant g14-3-3, and each of the T214E and R200K mutants, likely as combination of both N-terminal and C-terminal dimerization. Moreover, faint bands at molecular weight compatible with pentameric forms were also visible suggesting the occurrence of higher oligomers that, although, could not be resolved in the gel matrix. In contrast, the formation of trimers and tetramers was disfavored in the T208A mutant, as well as in polyG10 mutant and, even more, in polyG20 mutant (Fig. 7B, left panel), thus suggesting that in these mutants C-terminal dimerization could be hampered. Furthermore, when the incubation and the cross-linking were performed in presence of DTT no differences in oligomerization could be observed (Fig. 7B, right panel), thus indicating that disulfide bridges, if any, are not involved in g14-3-3 oligomerization.

Similarly, in native electrophoresis both h14-3-3ζ and endogenous g14-3-3 existed exclusively as N-terminal dimers (Fig. 7C). Compatibly with the gel resolution limits, for the recombinant g14-3-3, and each of the T214E and R200K mutants, a tetrameric form was favored over the dimeric one, with the R200K mutant nearly completely tetrameric, as supported by densitometric analysis of the protein bands (Fig. 7C–7D). On the opposite, in the polyG20, polyG10 and T208A mutants, the dimeric form was prevalent, with polyG20≥polyG10≈T208A (Fig. 7C–7D), further supporting that C-terminal dimerization was disfavored. For all the tested proteins, binding to target did not affect neither the dimerization nor the oligomerization as it resulted by the incubation of the proteins alternatively with the Raf1p phosphopeptide, reproducing a g14-3-3 consensus mode-1 binding sequence of the Raf-1 protein kinase [15], or the corresponding non phosphorylated peptide (Raf1) (Fig.7B-7C). However, this observation cannot be extended to the endogenous g14-3-3 that, due to the purification procedure, was already in complex with the Raf1p (Fig. 7B–7C).

These results strongly indicate that: i) the addition of a C-terminal polyglycine stretch, but not the presence of a negative charge at position T214, primarily impairs oligomerization of recombinant g14-3-3; ii) mutations R200K and T208A indeed affect oligomerization but in opposite directions, i.e. R200K favors it whereas T208A does not.

**Figure 8:**
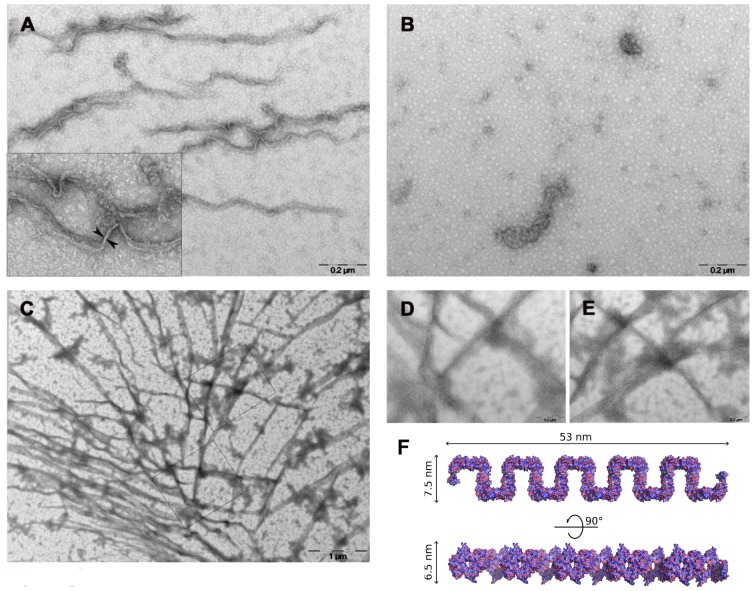
Recombinant g14-3-3 form filaments in a concentration-dependent manner. A) Recombinant g14-3-3 (0.1 μg/μl) forms numerous filaments with moderate length (between 0,8–1,6 μm) and an average diameter of 5,2±0,7 nm, as measured by iTEM soft imaging system (OLYMPUS). B) At the same concentration (0.1 μg/μl), the presence of the polyglycine stretch in the polyG20 mutant prevents filaments formation and the protein is found only as amorphous aggregates. C) Longer filaments (more than 3–4 μm) are formed by recombinant g14-3-3 at higher concentration (1 μg/μl). D) and E) are sections of panel C at high magnification (100.000X). Representative fields on the grids are depicted. F) Structure-based views of a g14-3-3 filament of concatenated 10 dimers. Dimension (nm) of the filaments are reported.

Finally, to further prove that the observed g14-3-3 filaments were not a crystallographic artifact and that recombinant g14-3-3 can form multimers in solution, the protein was studied with transmission electron microscopy. As shown (Fig. 8A), filaments of approximately 6 nm of thickness and ranging in length from 0,8 up to 1,6 μm were visible in the g14-3-3 samples. Intersecting filaments of greater length (up to 4 μm) were observed when the protein concentration was increased from 0.1 to 1 μg/μl (Fig. 8C, 8D and 8E). In agreement with native gel and cross-linking data, no filament could be observed in the polyG20 sample (Fig. 8B). The size of the observed g14-3-3 filaments was also in good agreement with measurement obtained from the solved crystal structure (Fig. 8F). Altogether, these data are in favor of the notion that oligomerization of g14-3-3 end up in filamentous structures, and non in globular structures, that according to the crystallographic data likely depends on domain swapping-mediated C-terminal dimerization.

## Discussion

We report the X-ray structure of *Giardia duodenalis* 14-3-3 (g14-3-3). A structural analysis confirmed that the protein has many of the features common to the other 14-3-3 family members and also highlights functional/structural roles for two peculiar g14-3-3 post-translational modifications, i.e. phosphorylation of Thr214 and polyglycylation of Glu246.

Despite the quite large number of 14-3-3 crystal structures available, we provide the first evidence that a *Giardia* member of the 14-3-3 family, deprived of post-translational modifications, forms oligomers *in vitro*. Analyses of the crystal structure revealed that recombinant unmodified g14-3-3 forms filaments likely by concatenation of the functional dimer. Such process, which was further confirmed by transmission electron microscopy, is enabled by the combination of N-terminal dimerization and the swapping of two helices between two adjacent g14-3-3 monomers with the formation of a four-helix bundle. Interestingly, this assembly retains the peptide-binding groove, but requires the rearrangement of the HI-loop with respect to other known 14-3-3 structures.

Domain swapping is dependent on the so called “hinge loop”, defined as a region of the protein that folds back on itself in the monomer but adopts an extended conformation in the domain-swapped form. Mutations in this region can in principle modulate the propensity of the protein to domain-swap (44). Consistently with this notion and our MD simulations, the g14-3-3 HI region seems to represent the hinge loop, since it hosts residues responsible for the “extended” C-terminal conformation, which are unique among 14-3-3 family members, such as Thr208 in the beginning of loop HI, and Arg200, in helix H. Indeed, mutation of Thr208 to an evolutionary conserved alanine and Arg200 to an evolutionary conserved lysine affects oligomerization but in opposite directions. As expected, T208A slightly decreases oligomerization likely due to the inability of alanine to form hydrogen bonds with Gln226 and Arg229 thus weakening the interactions between helices H and I. In contrast, the increased oligomerization due to the R200K mutation can be explained by the ability of the arginine to establish an intra-helix interaction with Asn233 and an inter-helix interaction with Asp204 (in helix H), thus favoring an equilibrium, whereas the lysine can only interact with Asn233 on the same helix (Fig. 3D).

The exclusively dimeric nature of the *in vivo* polyglycylated g14-3-3 and the hampered oligomerization and filament formation in both polyG10 and polyG20 mutants strongly suggest that the physiological role of polyglycylation is to prevent oligomerization of the protein *in vivo*. This is consistent with the observation that g14-3-3 is always polyglycylated *in vivo*. In fact, a very short polyglycine chain (less than ten residues) can be detectable at the g14-3-3 C-terminus even in parasite lines over-expressing the two g14-3-3-specific deglycylases [19], supporting a detrimental effect of the lack of the polyglycine chain *in vivo*. This role of polyglycylation is in addition to the previously described regulation of stage-dependent re-distribution of g14-3-3 between nuclei and cytoplasm already assigned to this PTM [15], [18]. The mechanism by which polyglycylation impairs oligomerization may be due to steric hindrance and/or disorder promotion. In this regard, the C-terminal tail of all known 14-3-3s is enriched in disorder-promoting residues (i.e. glycine). In fact, deletion of the last 15 C-terminal residues of the h14-3-3ζ induces aggregation of the protein in solution at physiological temperature, even if the protein retains its ability to bind phosphorylated ligands [45]. Since the g14-3-3 displays a shorter C-terminal tail, polyglycylation may introduce disorder in the protein [46]–[47]. Since there is evidence that polyglycine stretches can form extended structures both in crystals and in solution, we can speculate that an extended polyglycine chain could lay between helices H and I of each g14-3-3 monomer and act as a barrier hindering domain swapping. Intriguingly, in the C-terminal part of both the *S. cerevisiae* 14-3-3 isoforms, BMH1 and BMH2, is present a polyglutamine (polyQ) stretch (10 and 17 glutamine residues, respectively) and, when looking at protein alignment, the polyQ stretch of both BMHs starts approximately in correspondence to the polyglycylation site, Glu246, of g14-3-3 (Fig. 2) [15], [18]. As support to our proposed role for polyglycylation, it has been shown that the polyQ rich C-terminus of BMHs doesn't induce protein aggregation but adopts an open extended conformation, is responsible to increase the hydrodynamic radius of the BMH molecules, if compared with h14-3-3ζ, and it does not lay in the binding groove and cannot function as an autoinhibitory binding domain, as described for mammalian and plant isoform [48]. In previous works we showed that the T214E mutant displays an increased binding efficiency *in vitro* toward a subset of mode-1 phosphopeptide, if compared to the non-phosphorylated recombinant protein [18]. MD simulations show that the C-terminal tail of the phosphorylated protein displays a more rigid and “closed” conformation than the non phosphorylated one, but yet not sufficient to prevent oligomerization. Consistently, the T214E mutant, in which the side chain of the glutamic acid mimics the phosphate moiety of the phosphorylated threonine, is still able to oligomerize. This suggests that the primary role of the constitutive g14-3-3 phosphorylation *in vivo* is rather target binding. In MD simulations, upon phosphorylation of Thr214, helix I moves closer to helix H and causes Trp235, Asn231 and Leu223 to steadily face the phosphopeptide binding groove. Interestingly, these three residues correspond to Trp228, Asn231 and Leu216 (h14-3-3ε numbering), which in plant and mammalian 14-3-3s are directly involved in the binding to target peptides both in phosphorylation-dependent and –independent manner [8]. In contrast, in the non phosphorylated g14-3-3, the interaction of Trp235, Asn231 and Leu223 with the target proteins would first require a large conformational change re-positioning helix I closer to helix H or would need domain swapping of helix I' from another g14-3-3 dimer. This would likely entail a weaker initial binding of g14-3-3 to its target. Thus, *in vivo* phosphorylation might promote a faster and/or much more stable interaction with at least a pool of client proteins. This may also explain the defect in encystation observed when the non-phosphorylatable T214A mutant is overexpressed in *Giardia* [18].

The determination of the g14-3-3 structure raises new interesting questions. Due to the early evolutionary branching of *Giardia* and the long evolutionary history of this parasite, why have divergent residues been fixed in the g14-3-3 leading to conformational rearrangement and C-terminal domain swapping ? Why have PTMs been selected over amino acid mutations to overcame the risk of oligomerization? Completely deglycylated g14-3-3 has not been observed *in vivo* so far, however, it cannot be excluded that C-terminal domain swapping and limited oligomerization may occur *in vivo* possibly to mediate multiprotein complex formation. In this regard, the presence of 14-3-3s in protein aggregates have been observed in various human neurodegenerative diseases [49], although the biological significance of 14-3-3 in this context is still far to be clarified. Furthermore, arachidonic acid, known to favor tau and synuclein polymerization, also promotes *in vitro* oligomerization of h14-3-3ζ [50]. Intriguingly, sequence similarity at the C-terminal region, in particular in the HI loop, has also been observed between 14-3-3s [51]–[52], including g14-3-3 (data not shown) and α-synuclein, which form fibrils in Parkinson's diseases and co-localizes with perinuclear inclusions of hungtintin, and, thus strengthening the structural relevance of this region. It is tempting to speculate that ancestral 14-3-3s underwent C-terminal-mediated oligomerization and that this ability has been overcame during evolution in different ways, i.e. by post-translational polyglycylation, C-terminal polyglutamine stretch insertion, or engagement of the C-terminus tail into the binding groove.

In summary, we demonstrated that, despite the highly structural conservation of the 14-3-3 family members, unexpected features can be highlighted by the analysis of a 14-3-3 from the early branching eukaryote *Giardia duodenalis*. Since inhibition and/or stabilization of 14-3-3-protein interactions has gained substantial interest in pharmaceutical research and provided novel opportunity for the treatment of several diseases [53], the structural peculiarity of g14-3-3 may concretely support the possibility to design antigiardial drugs acting selectively as inhibitor or stabilizer of g14-3-3/target interaction, but ineffective on the human counterparts. The determination of the structure of phosphorylated and/or polyglycylated g14-3-3 in complex with binding peptides or protein targets would help answer several of the still open questions.

## Supporting Information

Figure S1 Detailed view of the electron density in the g14-3-3 phosphopeptide binding site. A) mFo-dFc omit map computed without sulfate ion, contoured at 3σ (green) or -3σ (red) in a radius of 4Å around the residues shown as sticks. B) mFo-dFc omit map as in Å plus 2mFo-dFc omit map (blue) contoured at 1σ. C) 2mFo-dFc (grey, 1σ) mFo-dFc (green, 3σ; red, -3σ) computed after sulfate ion modeling.
(JPG)

Figure S2 Tandem MS analysis of the g14-3-3 phosphorylated peptide (202-219). The MS/MS spectrum of the phosphopeptide A_202_FDAAITDLDKLpTEESYK_219_ (precursor ion (MH_2_)^2+^ 1055.5) is shown. Detected peaks corresponding to the ions of the *b* and *y* series are labeled and indicated in *red* and blue respectively. The ion at m/z 1006.6 is due to 49 Da neutral loss, corresponding to a phosphoric acid molecule, and demonstrates the presence of a phosphorylated peptide. The distance between y_5_ and y_6_ definitively localizes the phophorylation on the threonine 214. No peak corresponding to the unphosphorylated peptide could be observed.
(TIF)

## References

[1] Rosenquist M, Sehnke P, Ferl RJ, Sommarin M, Larsson C (2000) Evolution of the 14-3-3 protein family: does the large number of isoforms in multicellular organisms reflect functional specificity? J Mol Evol 51: 446–548. 11080367 10.1007/s002390010107

[2] Siles-Lucas M del M, Gottstein B (2003) The 14-3-3 protein: a key molecule in parasites as in other organisms. Trends Parasitol 19: 575–581. 14642768 10.1016/j.pt.2003.10.003

[3] Morrison DK (2009) The 14-3-3 proteins: integrators of diverse signaling cues that impact cell fate and cancer development. Trends Cell Biol 19: 16–23. 19027299 10.1016/j.tcb.2008.10.003 PMC3073487

[4] Liu D, Bienkowska J, Petosa C, Collier R J, Fu H, et al (1995) Crystal structure of the ζ isoform of the 14-3-3 protein. Nature 376: 191–194. 7603574 10.1038/376191a0

[5] Würtele M, Jelich-Ottmann C, Wittinghofer A, Oecking C (2003) Structural view of a fungal toxin acting on a 14-3-3 regulatory complex. EMBO J 22: 987–994. 12606564 10.1093/emboj/cdg104 PMC150337

[6] Gardino AK, Smerdon SJ, Yaffe MB (2006) Structural determinants of 14-3-3 binding specificities and regulation of subcellular localization of 14-3-3-ligand complexes: a comparison of the X-ray crystal structures of all human 14-3-3 isoforms. Semin Cancer Biol 16: 173–182. 16678437 10.1016/j.semcancer.2006.03.007

[7] Brokx SJ, Wernimont AK, Dong A, Wasney GA, Lin YH, et al (2011) Characterization of 14-3-3 proteins from Cryptosporidium parvum. PLoS One 6: e14827. 21853016 10.1371/journal.pone.0014827 PMC3154893

[8] Obsil T, Obsilova V (2011) Structural basis of 14-3-3 protein functions. Semin. Cell Dev Biol 22: 663–672. 10.1016/j.semcdb.2011.09.001 21920446

[9] Yaffe MB, Rittinger K, Volinia S, Caron PR, Aitken A, et al (1997) The structural basis for 14-3-3:phosphopeptide binding specificity. Cell 91: 961–971. 9428519 10.1016/s0092-8674(00)80487-0

[10] Coblitz B, Wu M, Shikano S, Li M (2006) C-terminal binding: an expanded repertoire and function of 14-3-3 proteins. FEBS Lett 580: 1531–1535. 16494877 10.1016/j.febslet.2006.02.014

[11] Aitken A (2006) 14-3-3 proteins: a historic overview. Semin Cancer Biol 16: 162–172. 16678438 10.1016/j.semcancer.2006.03.005

[12] Thompson RC (2000) Giardiasis as a re-emerging infectious disease and its zoonotic potential. Int. J. Parasitol. 12–13: 1259–1267. 10.1016/s0020-7519(00)00127-2 11113253

[13] Thompson RC, Monis P (2012) Giardia-from genome to proteome. Adv Parasitol 78: 57–95. 22520441 10.1016/B978-0-12-394303-3.00003-7

[14] Morrison HG, McArthur AG, Gillin FD, Aley SB, Adam RD, et al (2007) Genomic minimalism in the early diverging intestinal parasite Giardia lamblia. Science 317: 1921–1926. 17901334 10.1126/science.1143837

[15] Lalle M, Salzano AM, Crescenzi M, Pozio E (2006) The Giardia duodenalis 14-3-3 protein is post-translationally modified by phosphorylation and polyglycylation of the C-terminal tail. J Biol Chem 281: 5137–5148. 16368691 10.1074/jbc.M509673200

[16] Lalle M, Camerini S, Cecchetti S, Sayadi A, Crescenzi M, et al (2012) Interaction network of the 14-3-3 protein in the ancient protozoan parasite Giardia duodenalis. J Proteome Res 11: 2666–2683. 22452640 10.1021/pr3000199

[17] Aitken A (2011) Post-translational modification of 14-3-3 isoforms and regulation of cellular function. Semin Cell Dev Biol 22: 673–680. 21864699 10.1016/j.semcdb.2011.08.003

[18] Lalle M, Bavassano C, Fratini F, Cecchetti S, Boisguerin P, et al (2010) Involvement of 14-3-3 protein post-translational modifications in Giardia duodenalis encystation. Int J Parasitol 40: 201–213. 19733174 10.1016/j.ijpara.2009.07.010

[19] Lalle M, Camerini S, Cecchetti S, Blasetti Fantauzzi C, Crescenzi M, et al (2011) Giardia duodenalis 14-3-3 protein is polyglycylated by a tubulin tyrosine ligase-like member and deglycylated by two metallocarboxypeptidases. J Biol Chem 286: 4471–4484. 21135098 10.1074/jbc.M110.181511 PMC3039363

[20] Lalle M, Leptourgidou F, Camerini S, Pozio E, Skoulakis EMC (2013) Interkingdom complementation reveals structural conservation and functional divergence of 14-3-3 proteins. PLoS One 8(10): e78090. 24147113 10.1371/journal.pone.0078090 PMC3795638

[21] Tomassi L, Costantini A, Corallino S, Santonico E, Carducci M, et al (2008) The central proline rich region of POB1/REPS2 plays a regulatory role in epidermal growth factor receptor endocytosis by binding to 14-3-3 and SH3 domain-containing proteins. BMC Biochem 9: 21. 18647389 10.1186/1471-2091-9-21 PMC2494995

[22] Otwinowski Z, Minor W (1997) Processing of X-ray diffraction data collected in oscillation mode. Methods Enzymol 276: 307–326. 10.1016/S0076-6879(97)76066-X 27754618

[23] Collaborative Computational Project Number 4 (1994) The CCP4 Suite: Programs for Protein Crystallography. Acta Crystallogr D Biol Crystallogr 50: 760–763. 15299374 10.1107/S0907444994003112

[24] McCoy AJ, Grosse-Kunstleve RW, Adams PD, Winn MD, Storoni LC, et al (2007) Phaser crystallographic software. J Appl Crystallogr 40: 658–674. 19461840 10.1107/S0021889807021206 PMC2483472

[25] Yang X, Lee WH, Sobott F, Papagrigoriou E, Robinson CV, et al (2006) Structural basis for protein-protein interactions in the 14-3-3 protein family. Proc Natl Acad Sci USA 103: 17237–17242. 17085597 10.1073/pnas.0605779103 PMC1859916

[26] Emsley P, Cowtan K (2004) Coot: model-building tools for molecular graphics. Acta Crystallogr D Biol Crystallogr 60: , 2126–2132. 10.1107/S0907444904019158 15572765

[27] Murshudov GN, Vagin AA, Dodson EJ (1997) Refinement of macromolecular structures by the maximum-likelihood method. Acta Crystallogr D Biol Crystallogr 53: , 240–255. 10.1107/S0907444996012255 15299926

[28] DeLano WL (2002) The PyMOL Molecular Graphics System. http://www.pymol.org.

[29] Krissinel E, Henrick K (2007) Inference of macromolecular assemblies from crystalline state. J Mol Biol 372: 774–797. 17681537 10.1016/j.jmb.2007.05.022

[30] Krissinel E, Henrick K (2004) Secondary-structure matching (SSM), a new tool for fast protein structure alignment in three dimensions. Acta Crystallogr D Biol Crystallogr 60: 2256–2268. 15572779 10.1107/S0907444904026460

[31] Phillips JC, Braun R, Wang W, Gumbart J, Tajkhorshid E, et al (2005) Scalable molecular dynamics with NAMD. J Comput Chem 26: 1781–1802. 16222654 10.1002/jcc.20289 PMC2486339

[32] Jorgensen WL, Chandrasekhar J, Madura JD, Impey RW, Klein ML (1983) Comparison of simple potential functions for simulating liquid water. J Chem Phys 79: 926–935.

[33] Brooks BR, Bruccoleri RE, Olafson BD, States DJ, Swaminathan S, et al (1983) CHARMM: A Program for Macromolecular Energy, Minimization, and Dynamics Calculations. J Comput Chem 4: 187–217.

[34] Verlet L (1967) Computer experiments on classical fluids. I. Thermodynamical properties of Lennard-Jones molecules. Phys Rev 159: 98–103.

[35] Hess B, Bekker H, Berendsen HJC, Fraaije J (1997) LINCS: A linear constraint solver for molecular simulations. J Comput Chem 18: 1463–1472.

[36] Darden T, York D, Pedersen L (1993) Particle mesh Ewald: An N (center-dot) log(N) method for Ewald sums in large systems. J Chem Phys 98: 10089–10092.

[37] Arfken G (1985) The Method of Steepest Descents. §7.4 in Mathematical Methods for Physicists, 3rd ed. Orlando: Academic Press. pp. 428–436.

[38] Parrinello M, Rahman A (1981) Polymorphic transitions in single crystals: A new molecular dynamics method. J Appl Phys 52: 7182–7190.

[39] Pronk S, Páll S, Schulz R, Larsson P, Bjelkmar P, et al (2013) GROMACS 4.5: a high-throughput and highly parallel open source molecular simulation toolkit. Bioinformatics 29: 845–54. 23407358 10.1093/bioinformatics/btt055 PMC3605599

[40] Garcia AE (1992) Large-amplitude nonlinear motions in proteins. Phys Rev Lett 68: 2696–2699. 10045464 10.1103/PhysRevLett.68.2696

[41] Pettersen EF, Goddard TD, Huang CC, Couch GS, Greenblatt DM, et al (2004) UCSF Chimera–a visualization system for exploratory research and analysis. J Comput Chem 25: 1605–1612. 15264254 10.1002/jcc.20084

[42] Haladová K, Mrázek H, Ječmen T, Halada P, Man P, et al (2012) The combination of hydrogen/deuterium exchange or chemical cross-linking techniques with mass spectrometry: mapping of human 14-3-3ζ homodimer interface. J Struct Biol 179: 10–17. 22580067 10.1016/j.jsb.2012.04.016

[43] Wan WY, Milner-White EJ (1999) A recurring two-hydrogen-bond motif incorporating a serine or threonine residue is found both at alpha-helical N termini and in other situations. J Mol Biol 286: 1651–1662. 10064721 10.1006/jmbi.1999.2551

[44] Rousseau F, Schymkowitz J, Itzhaki LS (2012) Implications of 3D domain swapping for protein folding, misfolding and function. Adv Exp Med Biol 747: 137–52. 22949116 10.1007/978-1-4614-3229-6_9

[45] Williams DM, Ecroyd H, Goodwin KL, Dai H, Fu H, et al (2011) NMR spectroscopy of 14-3-3ζ reveals a flexible C-terminal extension: differentiation of the chaperone and phosphoserine-binding activities of 14-3-3ζ. Biochem J 437: 493–503. 21554249 10.1042/BJ20102178

[46] Lotz B (1974) Crystal structure of polyglycine I. J Mol Biol 87: 169–180. 4427364 10.1016/0022-2836(74)90141-7

[47] Ohnishi S, Kamikubo H, Onitsuka M, Kataoka M, Shortle D (2006) Conformational preference of polyglycine in solution to elongated structure. J Am Chem Soc 128: 16338–16344. 17165789 10.1021/ja066008b

[48] Veisova D, Rezabkova L, Stepanek M, Novotna P, Herman P, et al (2010) The C-terminal segment of yeast BMH proteins exhibits different structure compared to other 14-3-3 protein isoforms. Biochemistry 49: 3853–3861. 20384366 10.1021/bi100273k

[49] Steinacker P, Aitken A, Otto M (2011) 14-3-3 proteins in neurodegeneration. Semin Cell Dev Biol 22: 696–704. 21920445 10.1016/j.semcdb.2011.08.005

[50] Brock TG (2008) Arachidonic acid binds 14-3-3zeta, releases 14-3-3zeta from phosphorylated BAD and induces aggregation of 14-3-3zeta. Neurochem Res 33: 801–807. 17940884 10.1007/s11064-007-9498-3

[51] Ostrerova N, Petrucelli L, Farrer M, Mehta N, Choi P, et al (1999) Alpha-Synuclein shares physical and functional homology with 14-3-3 proteins. J Neurosci 19: 5782–5791. 10407019 10.1523/JNEUROSCI.19-14-05782.1999 PMC6783081

[52] Kim TD, Choi E, Rhim H, Paik SR, Yang CH (2004) Alpha-synuclein has structural and functional similarities to small heat shock proteins. Biochem Biophys Res Commun 324: 1352–1359. 15504363 10.1016/j.bbrc.2004.09.208

[53] Ottmann C (2013) Small-molecule modulators of 14-3-3 protein-protein interactions. Bioorg Med Chem 21: 4058–4062. 23266179 10.1016/j.bmc.2012.11.028

