# Supplementary figures and images for: The Crystal Structure of Giardia duodenalis 14-3-3 in the Apo Form: When Protein Post-Translational Modifications Make the Difference
Source: PLoS One. 2014 Mar 21;9(3):e92902. doi: 10.1371/journal.pone.0092902 (PMC3962474; doi:10.1371/journal.pone.0092902)

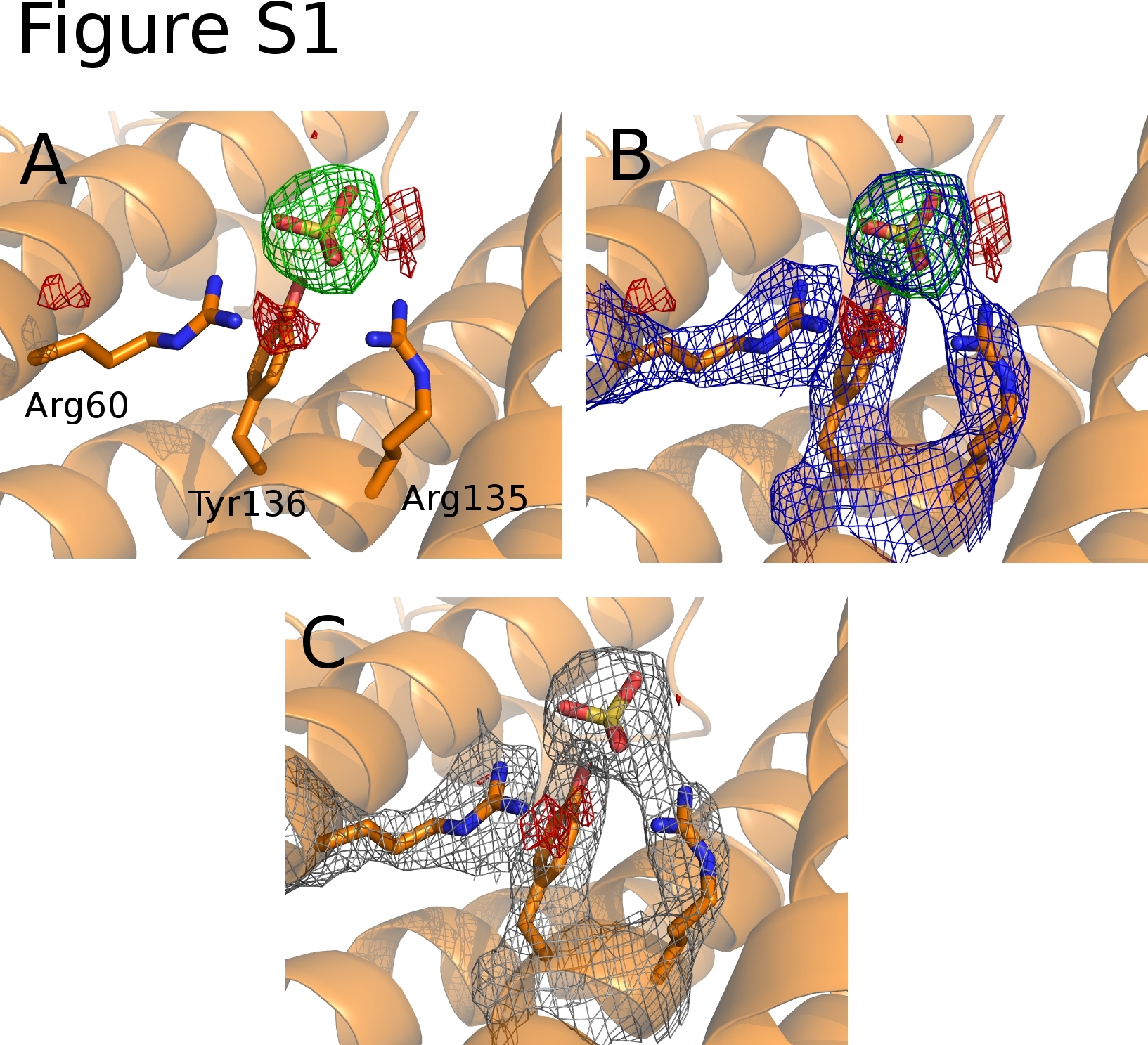

Supplement: Figure S1 — Detailed view of the electron density in the g14-3-3 phosphopeptide binding site. A) mFo-dFc omit map computed without sulfate ion, contoured at 3σ (green) or -3σ (red) in a radius of 4Å around the residues shown as sticks. B) mFo-dFc omit map as in Å plus 2mFo-dFc omit map (blue) contoured at 1σ. C) 2mFo-dFc (grey, 1σ) mFo-dFc (green, 3σ; red, -3σ) computed after sulfate ion modeling. (JPG) [file pone.0092902.s001.jpg]

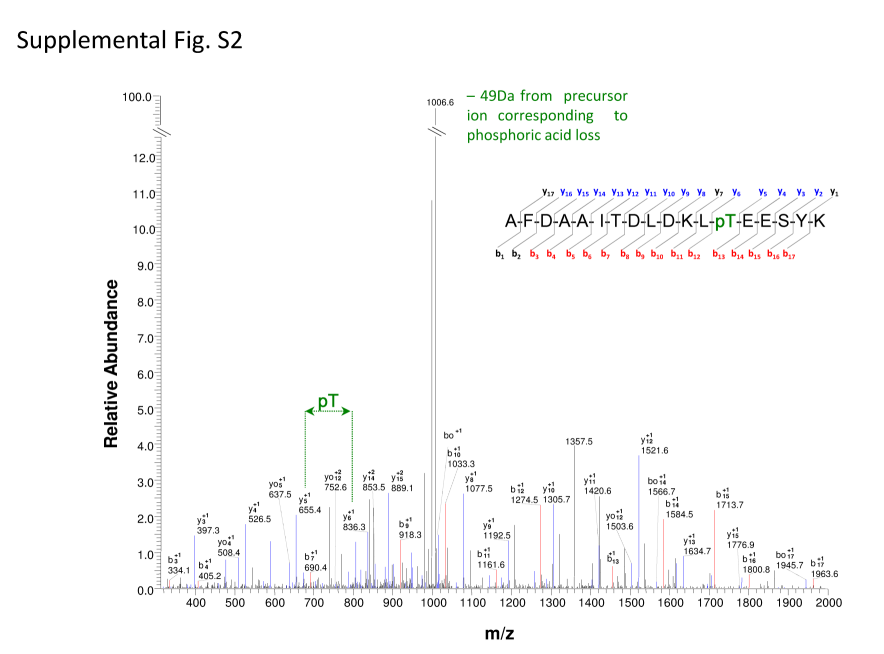

Supplement: Figure S2 — Tandem MS analysis of the g14-3-3 phosphorylated peptide (202-219). The MS/MS spectrum of the phosphopeptide A202FDAAITDLDKLpTEESYK219 (precursor ion (MH2)2+ 1055.5) is shown. Detected peaks corresponding to the ions of the b and y series are labeled and indicated in red and blue respectively. The ion at m/z 1006.6 is due to 49 Da neutral loss, corresponding to a phosphoric acid molecule, and demonstrates the presence of a phosphorylated peptide. The distance between y5 and y6 definitively localizes the phophorylation on the threonine 214. No peak corresponding to the unphosphorylated peptide could be observed. (TIF) [file pone.0092902.s002.tif]
